# A Permutation Test for Oligoset DNA Pooling Studies

**DOI:** 10.1371/journal.pone.0119096

**Published:** 2015-03-12

**Authors:** Hsiao-Yuan Huang, Jui-Hsiang Lin, Wen-Chung Lee

**Affiliations:** Research Center for Genes, Environment and Human Health, and Institute of Epidemiology and Preventive Medicine, College of Public Health, National Taiwan University, Taipei, Taiwan; Ohio State University Medical Center, UNITED STATES

## Abstract

Case-control association studies often suffer from population stratification bias. A previous triple combination strategy of stratum matching, genomic controlling, and multiple DNA pooling can correct the bias and save genotyping cost. However the method requires researchers to prepare a multitude of DNA pools—more than 30 case-control pooling sets in total (polyset). In this paper, the authors propose a permutation test for oligoset DNA pooling studies. Monte-Carlo simulations show that the proposed test has a type I error rate under control and a power comparable to that of individual genotyping. For a researcher on a tight budget, oligoset DNA pooling is a viable option.

## Introduction

Case-control association studies often suffer from population stratification bias [1–4]. Huang and Lee [5] recently proposed a triple combination strategy, which combines stratum matching, genomic controlling, and multiple DNA pooling. The strategy can correct population stratification bias and save genotyping cost.

Huang and Lee’s method [5] is a large-sample method for *polyset* DNA pooling studies, requiring researchers to prepare a multitude of DNA pools—more than 30 case-control pooling sets in totals. This may be impractical for most DNA pooling studies. Here we propose a permutation test for *oligoset* DNA pooling studies—as few as 10 pooling sets suffice. We use simulated and real data to demonstrate our method.

## Methods

Assume that there are a total of *n* cases recruited in the study. For each case, *m* (*m* ≥ 1) stratum-matched control(s) are also recruited (based on stratum-delineating variables). The multiple DNA pooling strategy is performed to construct a total of *J*(*j* = 1,…,*J*) pooling sets. Here we assume *J* < 30. A case with his/her matched control(s) is randomly allocated to one of the *J* pooling sets. In each and every pooling set, all the cases are pooled into a case pool, and the controls, into *m* control pool(s), making the total number of DNA pools of the study to be *J* × (1 + *m*). Next, the genomic control method is performed. Aside from the candidate marker of interest (*i* = 0), we randomly select a total of *L*(*i* = 1,…,*L*) null markers from the genome which are unlinked to or in linkage equilibrium with the candidate marker. The quantitative PCR is used for measuring the allele frequencies of the candidate marker and null markers for each pool. In the *j*th pooling set, the allele frequency for the *i*th marker for the case pool is labeled as *p*_1*ij*_, and the average allele frequency for the *m* control pool(s), as *p*_0*ij*._ We then calculate the test statistics for the candidate marker and all the null makers (*i* = 0,1,2,…,*L*): $\chi_{i}^{2}={\left(\overset{J}{\underset{j=1}{\sum }}D_{ij}\right)}^{2}/\overset{J}{\underset{j=1}{\sum }}D_{ij}^{2},$where *D*_*ij*_ = *p*_1*ij*_-*p*_0*ij*_. Finally, for correcting the residual population stratification bias, Huang and Lee’s [5] disequilibrium test statistic for the candidate marker is calculated: $T=\chi_{0}^{2}/mean\left\{\chi_{1}^{2},\;...,\chi_{L}^{2}\right\}.$

Because the total number of pooling set is small (*J* < 30), here we use permutation method to approximate the null sampling distribution of *T*. To be precise, we randomly shuffle the disease status in each pooling set and leave the genetic data unchanged. This can be achieved using a simple algorithm that multiply each and every column of the original data matrix *D*_*ij*_ randomly by +1 (disease status unchanged) or -1 (disease status exchanged). Based on this reshuffled data matrix, we then calculate a new *T* statistic for the candidate marker. The procedure is to be repeated a number of times, say, a total of 10000. A permutation p-value can be calculated as the proportion of the permutation *T* statistics larger than the *T* statistic of the original data.

## Results

### Simulation Study

Monte Carlo simulations were performed to examine the statistical properties of the permutation test. Here we follow the same simulation settings as in Huang and Lee’s paper [5], except that the number of pooling sets is small (*J* = 10, 15, and 25, respectively). The total number of cases is 900 and the total number of matched controls is 900 (*m* = 1) or 1800 (*m* = 2). The study population is assumed to be composed of a total of five hidden strata. The index of stratum mismatch, *δ* (0 ≤ *δ* ≤ 1), implies that a control is a random match with a probability of *δ*, and a perfect match with a probability of 1 - *δ*, to the case [5, 6]. The systematic error of the quantitative PCR measurement of DNA pools (unequal allelic amplification) was simulated by drawing a random *κ* value between 1 and 2 for each of the markers [5]. The measured allele frequency from the quantitative PCR is assumed to follow a logic normal distribution with a measurement error of *σ*. Ten thousand simulations were performed for each scenario. R codes for simulating data are given in S1 Exhibit.

**Fig 1:**
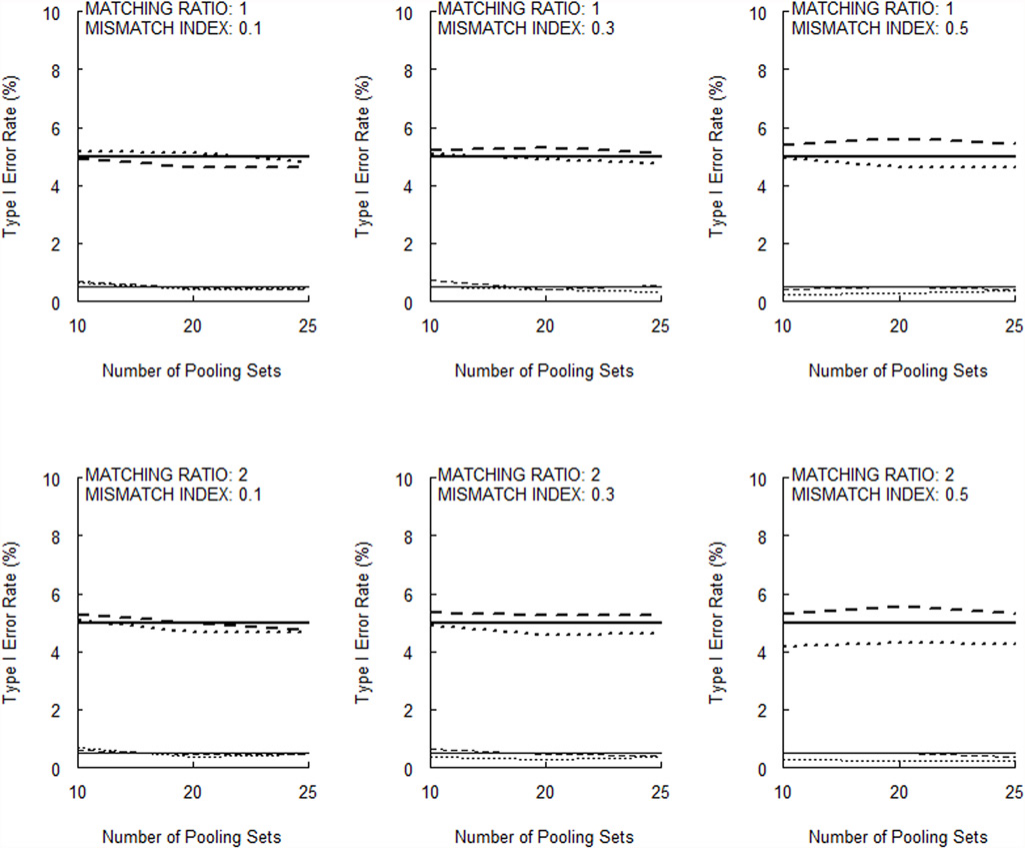
Type I error rates of the permutation test with a total of 10 null markers (bold broken lines, *σ* = 0.05, *α* = 0.05; thin broken lines, *σ* = 0.05, *α* = 0.005; bold dotted lines, *σ* = 0.01, *α* = 0.05; thin dotted lines, *σ* = 0.01, *α* = 0.005). The horizontal bold and thin solid lines indicate the nominal α level for *α* = 0.05 and *α* = 0.005, respectively.

**Fig 2:**
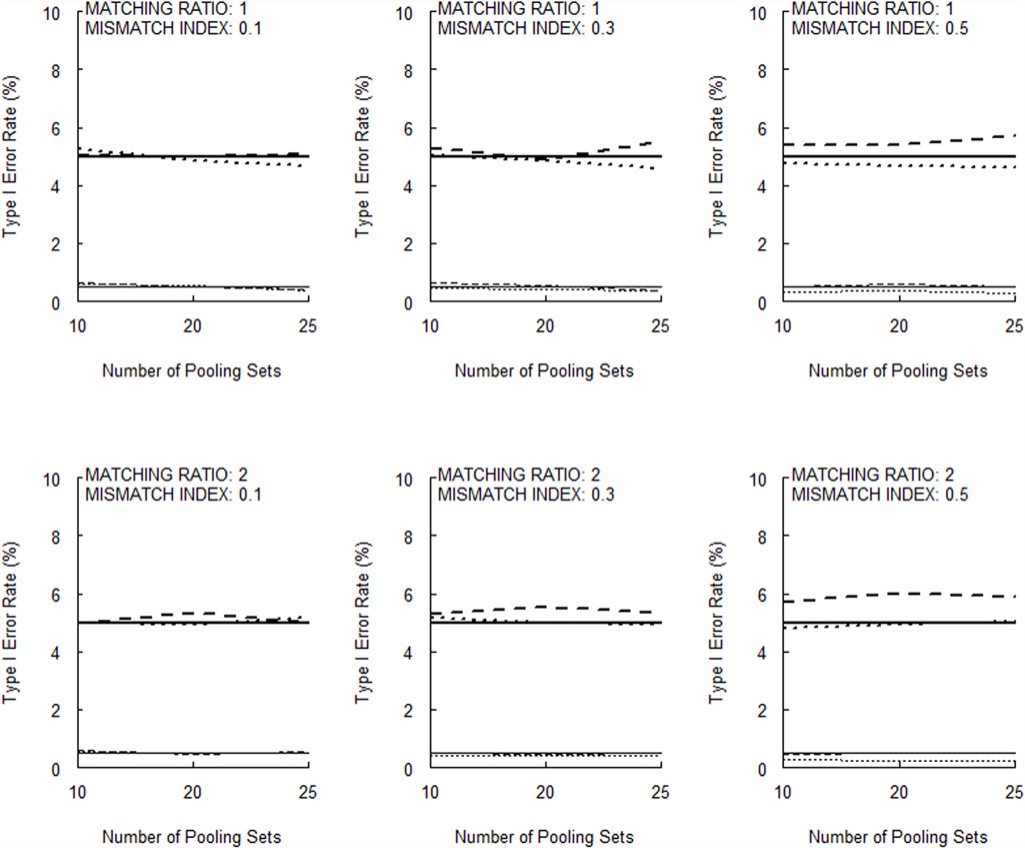
Type I error rates of the permutation test with a total of 50 null markers (bold broken lines, *σ* = 0.05, *α* = 0.05; thin broken lines, *σ* = 0.05, *α* = 0.005; bold dotted lines, *σ* = 0.01, *α* = 0.05; thin dotted lines, *σ* = 0.01, *α* = 0.005). The horizontal bold and thin solid lines indicate the nominal α level for *α* = 0.05 and *α* = 0.005, respectively.

Fig. 1 (for 10 null markers) and fig. 2 (for 50 null markers) show that the type I error rates of the permutation test are very close to the corresponding nominal *α* levels. S2 Exhibit and S3 Exhibit present the corresponding results when Huang and Lee’s [5] large-sample disequilibrium test is used instead. The conservatism in type I error rates is quite evident.

**Fig 3:**
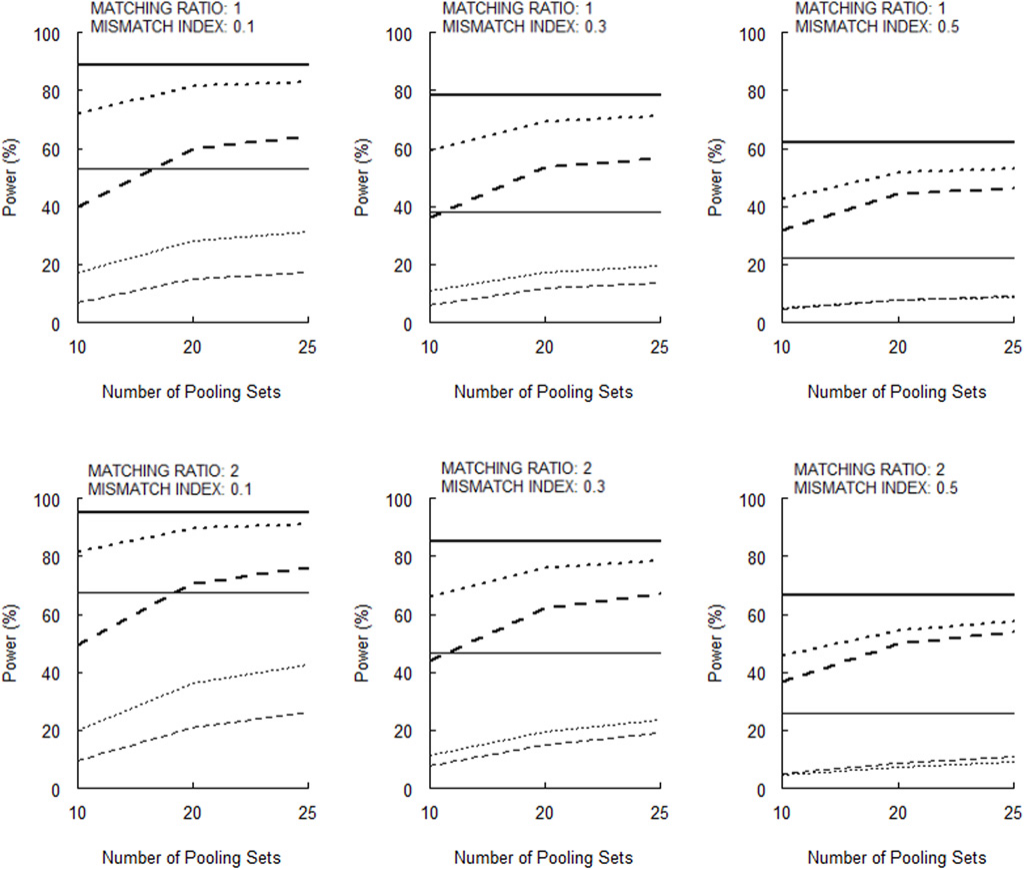
Powers of the permutation test with a total of 10 null markers (bold broken lines, *σ* = 0.05, *α* = 0.05; thin broken lines, *σ* = 0.05, *α* = 0.005; bold dotted lines, *σ* = 0.01, *α* = 0.05; thin dotted lines, *σ* = 0.01, *α* = 0.005). The horizontal bold and thin solid lines indicate the powers for the individual genotyping with stratum matching and genomic control for *α* = 0.05 and *α* = 0.005, respectively.

**Fig 4:**
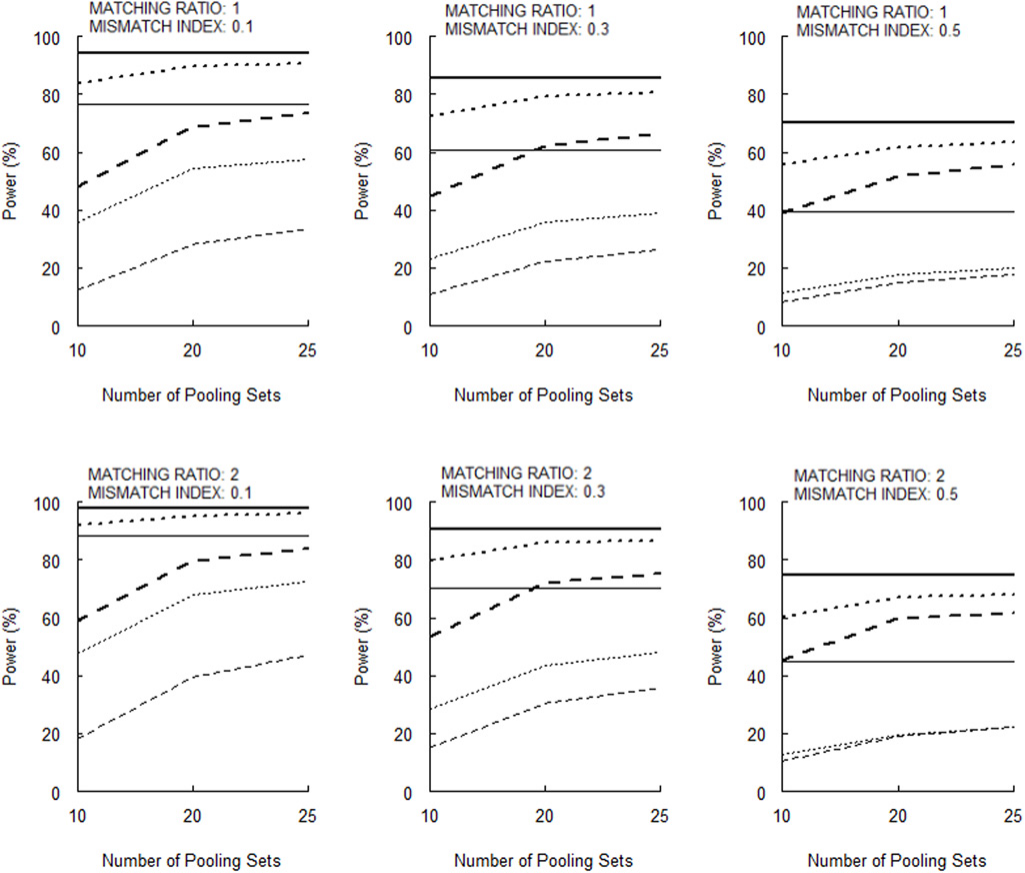
Powers of the permutation test with a total of 50 null markers (bold broken lines, *σ* = 0.05, *α* = 0.05; thin broken lines, *σ* = 0.05, *α* = 0.005; bold dotted lines, *σ* = 0.01, *α* = 0.05; thin dotted lines, *σ* = 0.01, *α* = 0.005). The horizontal bold and thin solid lines indicate the powers for the individual genotyping with stratum matching and genomic control for *α* = 0.05 and *α* = 0.005, respectively.

Fig. 3 (for 10 null markers) and fig. 4 (for 50 null markers) show that the power for the permutation test increases as the number of pooling sets increases. These Figures also show that the power is larger for a larger matching ratio (*m* = 2 vs. 1), more null markers in genomic control (50 vs. 10), smaller measurement error (*σ* = 0.01 vs. 0.05), and lower mismatch index (cf., 0.1, 0.3, and 0.5). [When Huang and Lee’s [5] large-sample disequilibrium test is used, the powers are lower (S4 Exhibit and S5 Exhibit).] For a stratum-matched case-control study with a mismatch index of 0.1, the permutation test of a DNA pooling with 25 pooling sets and a measurement error of 0.01 can have a power that is comparable to that when an individual genotyping was performed (horizontal solid lines in figs. 3 and 4).

### Real Data Example

We used Yamada et al.’s data [7] to demonstrate our method. The data consists of the genotypes of a total of 120 schizophrenia patients in Japan and their parents. Here we focus on one marker, rs2174623 at 4q28.1, which has a very significant p-value of 6.11×10^-6^ with individual genotyping.

For genomic control, we randomly chose a total of 10 and 50 null markers, respectively, from across the genome. To study the effect of DNA pooling, we formed a total of 10, 12, 15, 20, and 24 pooling sets, respectively. Each case together with his/her parents is randomly assigned to one of the pooling sets. At each pooling set, the cases are pooled into a single ‘case pool’, the fathers, a single ‘father pool’, and the mothers, a single ‘mother pool’. (Note that a case-parent study, such as Yamada et al.’s, is essentially a 1:2 stratum-matched case-control study [8].) We simulated the unequal allelic amplifications and measurement errors for this dataset the same way as in the previous simulation study section.

Table 1 showed that the p-values of the permutation test are significant (at *α* = 0.05) for all scenarios. The p-values are smaller for more null markers in genomic control (50 vs. 10), smaller measurement error (*σ* = 0.01 vs. 0.05), and more pooling sets used. The permutation test of a DNA pooling with 24 pooling sets, 50 null markers for genomic control, and a measurement error of 0.01, can have a p-value of 2.25×10^-5^ which is close to the p-value of 6.11×10^-6^ reported in Yamada’s paper [7].

**Table 1:** The results of a permutation test for oligoset DNA pooling studies for the example data.

Number of pooling sets	10 null markers		50 null markers	
	*σ* = 0.01	*σ* = 0.05	*σ* = 0.01	*σ* = 0.05
10	1.66×10^-2^	6.54×10^-2^	3.67×10^-3^	3.71×10^-2^
12	5.56×10^-3^	3.33×10^-2^	1.14×10^-3^	1.34×10^-2^
15	5.51×10^-3^	2.26×10^-2^	6.88×10^-4^	7.25×10^-3^
20	3.94×10^-3^	1.73×10^-2^	5.65×10^-5^	3.72×10^-3^
24	1.35×10^-3^	8.90×10^-3^	2.25×10^-5^	1.42×10^-3^

## Discussion

For a researcher on a tight budget, the triple combination strategy of stratum matching, genomic controlling, and oligoset DNA pooling is a viable design option. As shown in this paper, the permutation test has a type I error rate under control. This means that the all-in-one design by itself is a legitimate method for testing marker-disease association. This is in contrast to other *two-stage* (or *multi-stage*) designs, where the results from the first-stage DNA pooling need to be validated in the second-stage (or later-stage) individual genotyping studies [9–12]. Therefore our *one-stage* oligoset DNA pooling design can save cost tremendously. For example, for a ten-pooling-set case-control study with a total of 9000 cases and 9000 controls, only 10/9000 = 1/900 typing efforts are needed (without the need for any additional individual typing). Of course, if a researcher opts for high power more than low cost, he/she can perform polyset DNA pooling [5] or even dispense with the pooling procedure altogether [6]. But from our simulation study, there is a diminishing return in power as the number of pooling sets increases.

The associations between common variants and complex diseases are often very weak [13,14], although taken together, the small effects of all common variants may explain a larger (but not all) part of genetic components for common diseases [15,16]. Recently, more and more rare variants are being sequenced by next generation sequencing hopefully to account for the missing heritability [17,18]. To this end, many analyzing methods have been proposed [19], some of which are also using DNA pooling [20–23]. Further studies are warranted to extend the triple combination methods in this paper for use in rare-variant settings.

## Supporting Information

S1 Exhibit R code for simulating data. (DOC)

S2 Exhibit Type I error rates of Huang and Lee’s [5] large-sample disequilibrium test with a total of 10 null markers. (DOC)

S3 Exhibit Type I error rates of Huang and Lee’s [5] large-sample disequilibrium test with a total of 50 null markers. (DOC)

S4 Exhibit Powers of Huang and Lee’s [5] large-sample disequilibrium test with a total of 10 null markers. (DOC)

S5 Exhibit Powers of Huang and Lee’s [5] large-sample disequilibrium test with a total of 50 null markers. (DOC)
