# Supplementary material for: A Permutation Test for Oligoset DNA Pooling Studies
Source: PLoS One. 2015 Mar 12;10(3):e0119096. doi: 10.1371/journal.pone.0119096 (PMC4357378; doi:10.1371/journal.pone.0119096)
Supplement: S1 Exhibit — (DOC) [file pone.0119096.s001.doc]

**S1 Exhibit.** R code for simulating data.

We provide four scenarios of simulating setting here. There are the scenarios under the null hypothesis with matching ratio equal to one, under the null hypothesis with matching ratio equal to two, under the alternative hypothesis with matching ratio equal to one, and under the alternative hypothesis with matching ratio equal to two, respectively.

**Null hypothesis: Matching ratio =1**

# Stratum frequency

S_freq <- c(0.02,0.08,0.10,0.30,0.50)

# Disease prevalence in stratum s

Dis_prevalence <- c(4*10^-5,16*10^-5,8*10^-5,32*10^-5,2*10^-5)

# Case frequency in each stratum

Case_prevalence <- S_freq*Dis_prevalence

Case_frequency <- Case_prevalence/sum(Case_prevalence)

# F value in stratum s

F_strata <- c(0.008,0.006,0.010,0.012,0.014)

# Number of null markers

Null_markers <- 10

# Number of pooling sets

J_set <- 10

# Measurement error

M_error <- 0.01

# Mismatch index

Mism_index <- 0.1

# Simulation settings

Simulation_times <- 10000

Permutation_times <- 10000

Count <- 0

P_all <- NULL

ContProb <- rbind(

c(1-Mism_index+Mism_index*0.02,Mism_index*0.08,Mism_index*0.10,Mism_index*0.30,Mism_index*0.50),

c(Mism_index*0.02,1-Mism_index+Mism_index*0.08,Mism_index*0.10,Mism_index*0.30,Mism_index*0.50),

c(Mism_index*0.02,Mism_index*0.08,1-Mism_index+Mism_index*0.10,Mism_index*0.30,Mism_index*0.50),

c(Mism_index*0.02,Mism_index*0.08,Mism_index*0.10,1-Mism_index+Mism_index*0.30,Mism_index*0.50),

c(Mism_index*0.02,Mism_index*0.08,Mism_index*0.10,Mism_index*0.30,1-Mism_index+Mism_index*0.50)

)

for (z in 1:Simulation_times){

# Case sampling

Case_number <- 900

Case <- sample(1:5,Case_number,rep=T,prob=Case_frequency)

# Control sampling

Control_group1 <- rep(0,length(Case))

for(i in 1:5){

NCase <- sum(Case == i)

Control_group1[Case == i] <- sample(1:5,NCase,rep=T,prob=ContProb[i,])

}

allele_fi <- c(0.30,runif(Null_markers,0.05,0.95))

# Parameters of beta distribution

# Beta_alpha <- allele_fi*(1-F_strata)/F_strata

# Beta_beta <- (1-allele_fi)*(1-F_strata)/F_strata

allele_fis <- NULL

for (i in 1:length(F_strata)){

Beta_alpha <- allele_fi*(1-F_strata[i])/F_strata[i]

Beta_beta <- (1-allele_fi)*(1-F_strata[i])/F_strata[i]

allele_fistrata <- rbeta(length(allele_fi),Beta_alpha,Beta_beta)

allele_fis <- rbind(allele_fis,allele_fistrata)

}

Nallele_fis <- dim(allele_fis)[2]

# Genotypes

# Case marker

Case_marker <- (runif(Nallele_fis*Case_number) < allele_fis[Case,]) +

(runif(Nallele_fis*Case_number) < allele_fis[Case,])

# Control 1 marker

Group1_marker <- (runif(Nallele_fis*Case_number) < allele_fis[Control_group1,]) +

(runif(Nallele_fis*Case_number) < allele_fis[Control_group1,])

# Pooling sets

# Quantitative PCR

Pooling_set <- rep(1:J_set,each=900/J_set)

Case_pool <- cbind(Case,Pooling_set,Case_marker)

Case_pool <- data.frame(Case_pool)

Kappa <- runif(Nallele_fis,1,2)

Case_PCR_freq <- NULL

for (i in 1:J_set){

Each_set <- Case_marker[Pooling_set==i,]

EachSum <- colSums(Each_set)

PCR_freq <- EachSum*Kappa/(EachSum*(Kappa-1)+(900/J_set)*2)

PCR_freq <- exp(rnorm(Nallele_fis,PCR_freq,M_error))

PCR_freq <- PCR_freq / (1+PCR_freq)

Case_PCR_freq <- rbind(Case_PCR_freq,PCR_freq)

}

Group1_pool <- cbind(Control_group1,Pooling_set,Group1_marker)

Group1_pool <- data.frame(Group1_pool)

Group1_PCR_freq <- NULL

for (i in 1:J_set){

Each_set <- Group1_marker[Pooling_set==i,]

EachSum <- colSums(Each_set)

PCR_freq <- EachSum*Kappa/(EachSum*(Kappa-1)+(900/J_set)*2)

PCR_freq <- exp(rnorm(Nallele_fis,PCR_freq,M_error))

PCR_freq <- PCR_freq / (1+PCR_freq)

Group1_PCR_freq <- rbind(Group1_PCR_freq,PCR_freq)

}

Group_PCR_freq <- Group1_PCR_freq

# Statistics

D_matrix <- Case_PCR_freq-Group_PCR_freq

#D_matrix <- data.frame(D_matrix)

ChiS_total <- colSums(D_matrix)^2 / colSums(D_matrix^2)

T_statistics <- ChiS_total[1]/mean(ChiS_total[-1])

T_permutation <- NULL

for (i in 1:Permutation_times){

Per_vector <- sample(c(-1,1),rep=T,J_set,prob=c(0.5,0.5))

D_per_matrix <- D_matrix*Per_vector

ChiS_per_total <- colSums(D_per_matrix)^2 / colSums(D_per_matrix^2)

T_per_statistics <- ChiS_per_total[1]/mean(ChiS_per_total[-1])

T_permutation <- c(T_permutation,T_per_statistics)

}

P_statistics <- length(T_permutation[T_permutation>T_statistics])/length(T_permutation)

P_all <- c(P_all,P_statistics)

Count <- Count+ (P_statistics<0.05)

}

**Null hypothesis: Matching ratio =2**

# Stratum frequency

S_freq <- c(0.02,0.08,0.10,0.30,0.50)

# Disease prevalence in stratum s

Dis_prevalence <- c(4*10^-5,16*10^-5,8*10^-5,32*10^-5,2*10^-5)

# Case frequency in each stratum

Case_prevalence <- S_freq*Dis_prevalence

Case_frequency <- Case_prevalence/sum(Case_prevalence)

# F value in stratum s

F_strata <- c(0.008,0.006,0.010,0.012,0.014)

# Number of null markers

Null_markers <- 10

# Number of pooling sets

J_set <- 10

# Measurement error

M_error <- 0.01

# Mismatch index

Mism_index <- 0.1

# Simulation settings

Simulation_times <- 10000

Permutation_times <- 10000

Count <- 0

P_all <- NULL

ContProb <- rbind(

c(1-Mism_index+Mism_index*0.02,Mism_index*0.08,Mism_index*0.10,Mism_index*0.30,Mism_index*0.50),

c(Mism_index*0.02,1-Mism_index+Mism_index*0.08,Mism_index*0.10,Mism_index*0.30,Mism_index*0.50),

c(Mism_index*0.02,Mism_index*0.08,1-Mism_index+Mism_index*0.10,Mism_index*0.30,Mism_index*0.50),

c(Mism_index*0.02,Mism_index*0.08,Mism_index*0.10,1-Mism_index+Mism_index*0.30,Mism_index*0.50),

c(Mism_index*0.02,Mism_index*0.08,Mism_index*0.10,Mism_index*0.30,1-Mism_index+Mism_index*0.50)

)

for (z in 1:Simulation_times){

# Case sampling

Case_number <- 900

Case <- sample(1:5,Case_number,rep=T,prob=Case_frequency)

# Control sampling

Control_group1 <- rep(0,length(Case))

for(i in 1:5){

NCase <- sum(Case == i)

Control_group1[Case == i] <- sample(1:5,NCase,rep=T,prob=ContProb[i,])

}

Control_group2 <- rep(0,length(Case))

for(i in 1:5){

NCase <- sum(Case == i)

Control_group2[Case == i] <- sample(1:5,NCase,rep=T,prob=ContProb[i,])

}

allele_fi <- c(0.30,runif(Null_markers,0.05,0.95))

# Parameters of beta distribution

# Beta_alpha <- allele_fi*(1-F_strata)/F_strata

# Beta_beta <- (1-allele_fi)*(1-F_strata)/F_strata

allele_fis <- NULL

for (i in 1:length(F_strata)){

Beta_alpha <- allele_fi*(1-F_strata[i])/F_strata[i]

Beta_beta <- (1-allele_fi)*(1-F_strata[i])/F_strata[i]

allele_fistrata <- rbeta(length(allele_fi),Beta_alpha,Beta_beta)

allele_fis <- rbind(allele_fis,allele_fistrata)

}

Nallele_fis <- dim(allele_fis)[2]

# Genotypes

# Case marker

Case_marker <- (runif(Nallele_fis*Case_number) < allele_fis[Case,]) +

(runif(Nallele_fis*Case_number) < allele_fis[Case,])

# Control 1 marker

Group1_marker <- (runif(Nallele_fis*Case_number) < allele_fis[Control_group1,]) +

(runif(Nallele_fis*Case_number) < allele_fis[Control_group1,])

# Control 2 marker

Group2_marker <- (runif(Nallele_fis*Case_number) < allele_fis[Control_group2,]) +

(runif(Nallele_fis*Case_number) < allele_fis[Control_group2,])

# Pooling sets

# Quantitative PCR

Pooling_set <- rep(1:J_set,each=900/J_set)

Case_pool <- cbind(Case,Pooling_set,Case_marker)

Case_pool <- data.frame(Case_pool)

Kappa <- runif(Nallele_fis,1,2)

Case_PCR_freq <- NULL

for (i in 1:J_set){

Each_set <- Case_marker[Pooling_set==i,]

EachSum <- colSums(Each_set)

PCR_freq <- EachSum*Kappa/(EachSum*(Kappa-1)+(900/J_set)*2)

PCR_freq <- exp(rnorm(Nallele_fis,PCR_freq,M_error))

PCR_freq <- PCR_freq / (1+PCR_freq)

Case_PCR_freq <- rbind(Case_PCR_freq,PCR_freq)

}

Group1_pool <- cbind(Control_group1,Pooling_set,Group1_marker)

Group1_pool <- data.frame(Group1_pool)

Group1_PCR_freq <- NULL

for (i in 1:J_set){

Each_set <- Group1_marker[Pooling_set==i,]

EachSum <- colSums(Each_set)

PCR_freq <- EachSum*Kappa/(EachSum*(Kappa-1)+(900/J_set)*2)

PCR_freq <- exp(rnorm(Nallele_fis,PCR_freq,M_error))

PCR_freq <- PCR_freq / (1+PCR_freq)

Group1_PCR_freq <- rbind(Group1_PCR_freq,PCR_freq)

}

Group2_pool <- cbind(Control_group2,Pooling_set,Group2_marker)

Group2_pool <- data.frame(Group2_pool)

Group2_PCR_freq <- NULL

for (i in 1:J_set){

Each_set <- Group2_marker[Pooling_set==i,]

EachSum <- colSums(Each_set)

PCR_freq <- EachSum*Kappa/(EachSum*(Kappa-1)+(900/J_set)*2)

PCR_freq <- exp(rnorm(Nallele_fis,PCR_freq,M_error))

PCR_freq <- PCR_freq / (1+PCR_freq)

Group2_PCR_freq <- rbind(Group2_PCR_freq,PCR_freq)

}

Group_PCR_freq <- (Group1_PCR_freq+Group2_PCR_freq)/2

# Statistics

D_matrix <- Case_PCR_freq-Group_PCR_freq

#D_matrix <- data.frame(D_matrix)

ChiS_total <- colSums(D_matrix)^2 / colSums(D_matrix^2)

T_statistics <- ChiS_total[1]/mean(ChiS_total[-1])

T_permutation <- NULL

for (i in 1:Permutation_times){

Per_vector <- sample(c(-1,1),rep=T,J_set,prob=c(0.5,0.5))

D_per_matrix <- D_matrix*Per_vector

ChiS_per_total <- colSums(D_per_matrix)^2 / colSums(D_per_matrix^2)

T_per_statistics <- ChiS_per_total[1]/mean(ChiS_per_total[-1])

T_permutation <- c(T_permutation,T_per_statistics)

}

P_statistics <- length(T_permutation[T_permutation>T_statistics])/length(T_permutation)

P_all <- c(P_all,P_statistics)

Count <- Count+ (P_statistics<0.05)

}

**Alternative hypothesis: Matching ratio =1**

# Stratum frequency

S_freq <- c(0.02,0.08,0.10,0.30,0.50)

# Disease prevalence in stratum s

Dis_prevalence <- c(4*10^-5,16*10^-5,8*10^-5,32*10^-5,2*10^-5)

# Case frequency in each stratum

Case_prevalence <- S_freq*Dis_prevalence

Case_frequency <- Case_prevalence/sum(Case_prevalence)

# F value in stratum s

F_strata <- c(0.008,0.006,0.010,0.012,0.014)

# Number of null markers

Null_markers <- 10

# Number of pooling sets

J_set <- 30

# Measurement error

M_error <- 0.01

# Mismatch index

Mism_index <- 0.3

# Risk parameter

Gamma <- 1.3

# Simulation settings

Simulation_times <- 10000

Permutation_times <- 10000

Count <- 0

P_all <- NULL

ContProb <- rbind(

c(1-Mism_index+Mism_index*0.02,Mism_index*0.08,Mism_index*0.10,Mism_index*0.30,Mism_index*0.50),

c(Mism_index*0.02,1-Mism_index+Mism_index*0.08,Mism_index*0.10,Mism_index*0.30,Mism_index*0.50),

c(Mism_index*0.02,Mism_index*0.08,1-Mism_index+Mism_index*0.10,Mism_index*0.30,Mism_index*0.50),

c(Mism_index*0.02,Mism_index*0.08,Mism_index*0.10,1-Mism_index+Mism_index*0.30,Mism_index*0.50),

c(Mism_index*0.02,Mism_index*0.08,Mism_index*0.10,Mism_index*0.30,1-Mism_index+Mism_index*0.50)

)

for (z in 1:Simulation_times){

# Case sampling

Case_number <- 900

Case <- sample(1:5,Case_number,rep=T,prob=Case_frequency)

# Control sampling

Control_group1 <- rep(0,length(Case))

for(i in 1:5){

NCase <- sum(Case == i)

Control_group1[Case == i] <- sample(1:5,NCase,rep=T,prob=ContProb[i,])

}

allele_fi <- c(0.30,runif(Null_markers,0.05,0.95))

# Parameters of beta distribution

# Beta_alpha <- allele_fi*(1-F_strata)/F_strata

# Beta_beta <- (1-allele_fi)*(1-F_strata)/F_strata

allele_fis <- NULL

for (i in 1:length(F_strata)){

Beta_alpha <- allele_fi*(1-F_strata[i])/F_strata[i]

Beta_beta <- (1-allele_fi)*(1-F_strata[i])/F_strata[i]

allele_fistrata <- rbeta(length(allele_fi),Beta_alpha,Beta_beta)

allele_fis <- rbind(allele_fis,allele_fistrata)

}

Nallele_fis <- dim(allele_fis)[2]

allele_fiscase <- allele_fis

allele_fiscase[,1] <- (allele_fiscase[,1]*Gamma)/((allele_fiscase[,1]*Gamma)+1-allele_fiscase[,1])

# Genotypes

# Case marker

Case_marker <- (runif(Nallele_fis*Case_number) < allele_fiscase[Case,]) +

(runif(Nallele_fis*Case_number) < allele_fiscase[Case,])

# Control 1 marker

Group1_marker <- (runif(Nallele_fis*Case_number) < allele_fis[Control_group1,]) +

(runif(Nallele_fis*Case_number) < allele_fis[Control_group1,])

# Pooling sets

# Quantitative PCR

Pooling_set <- rep(1:J_set,each=900/J_set)

Case_pool <- cbind(Case,Pooling_set,Case_marker)

Case_pool <- data.frame(Case_pool)

Kappa <- runif(Nallele_fis,1,2)

Case_PCR_freq <- NULL

for (i in 1:J_set){

Each_set <- Case_marker[Pooling_set==i,]

EachSum <- colSums(Each_set)

PCR_freq <- EachSum*Kappa/(EachSum*(Kappa-1)+(900/J_set)*2)

PCR_freq <- exp(rnorm(Nallele_fis,PCR_freq,M_error))

PCR_freq <- PCR_freq / (1+PCR_freq)

Case_PCR_freq <- rbind(Case_PCR_freq,PCR_freq)

}

Group1_pool <- cbind(Control_group1,Pooling_set,Group1_marker)

Group1_pool <- data.frame(Group1_pool)

Group1_PCR_freq <- NULL

for (i in 1:J_set){

Each_set <- Group1_marker[Pooling_set==i,]

EachSum <- colSums(Each_set)

PCR_freq <- EachSum*Kappa/(EachSum*(Kappa-1)+(900/J_set)*2)

PCR_freq <- exp(rnorm(Nallele_fis,PCR_freq,M_error))

PCR_freq <- PCR_freq / (1+PCR_freq)

Group1_PCR_freq <- rbind(Group1_PCR_freq,PCR_freq)

}

Group_PCR_freq <- Group1_PCR_freq

# Statistics

D_matrix <- Case_PCR_freq-Group_PCR_freq

#D_matrix <- data.frame(D_matrix)

ChiS_total <- colSums(D_matrix)^2 / colSums(D_matrix^2)

T_statistics <- ChiS_total[1]/mean(ChiS_total[-1])

T_permutation <- NULL

for (i in 1:Permutation_times){

Per_vector <- sample(c(-1,1),rep=T,J_set,prob=c(0.5,0.5))

D_per_matrix <- D_matrix*Per_vector

ChiS_per_total <- colSums(D_per_matrix)^2 / colSums(D_per_matrix^2)

T_per_statistics <- ChiS_per_total[1]/mean(ChiS_per_total[-1])

T_permutation <- c(T_permutation,T_per_statistics)

}

P_statistics <- length(T_permutation[T_permutation>T_statistics])/length(T_permutation)

P_all <- c(P_all,P_statistics)

Count <- Count+ (P_statistics<0.05)

}

**Alternative hypothesis: Matching ratio =2**

# Stratum frequency

S_freq <- c(0.02,0.08,0.10,0.30,0.50)

# Disease prevalence in stratum s

Dis_prevalence <- c(4*10^-5,16*10^-5,8*10^-5,32*10^-5,2*10^-5)

# Case frequency in each stratum

Case_prevalence <- S_freq*Dis_prevalence

Case_frequency <- Case_prevalence/sum(Case_prevalence)

# F value in stratum s

F_strata <- c(0.008,0.006,0.010,0.012,0.014)

# Number of null markers

Null_markers <- 10

# Number of pooling sets

J_set <- 30

# Measurement error

M_error <- 0.01

# Mismatch index

Mism_index <- 0.3

# Risk parameter

Gamma <- 1.3

# Simulation settings

Simulation_times <- 10000

Permutation_times <- 10000

Count <- 0

P_all <- NULL

ContProb <- rbind(

c(1-Mism_index+Mism_index*0.02,Mism_index*0.08,Mism_index*0.10,Mism_index*0.30,Mism_index*0.50),

c(Mism_index*0.02,1-Mism_index+Mism_index*0.08,Mism_index*0.10,Mism_index*0.30,Mism_index*0.50),

c(Mism_index*0.02,Mism_index*0.08,1-Mism_index+Mism_index*0.10,Mism_index*0.30,Mism_index*0.50),

c(Mism_index*0.02,Mism_index*0.08,Mism_index*0.10,1-Mism_index+Mism_index*0.30,Mism_index*0.50),

c(Mism_index*0.02,Mism_index*0.08,Mism_index*0.10,Mism_index*0.30,1-Mism_index+Mism_index*0.50)

)

for (z in 1:Simulation_times){

# Case sampling

Case_number <- 900

Case <- sample(1:5,Case_number,rep=T,prob=Case_frequency)

# Control sampling

Control_group1 <- rep(0,length(Case))

for(i in 1:5){

NCase <- sum(Case == i)

Control_group1[Case == i] <- sample(1:5,NCase,rep=T,prob=ContProb[i,])

}

Control_group2 <- rep(0,length(Case))

for(i in 1:5){

NCase <- sum(Case == i)

Control_group2[Case == i] <- sample(1:5,NCase,rep=T,prob=ContProb[i,])

}

allele_fi <- c(0.30,runif(Null_markers,0.05,0.95))

# Parameters of beta distribution

# Beta_alpha <- allele_fi*(1-F_strata)/F_strata

# Beta_beta <- (1-allele_fi)*(1-F_strata)/F_strata

allele_fis <- NULL

for (i in 1:length(F_strata)){

Beta_alpha <- allele_fi*(1-F_strata[i])/F_strata[i]

Beta_beta <- (1-allele_fi)*(1-F_strata[i])/F_strata[i]

allele_fistrata <- rbeta(length(allele_fi),Beta_alpha,Beta_beta)

allele_fis <- rbind(allele_fis,allele_fistrata)

}

Nallele_fis <- dim(allele_fis)[2]

allele_fiscase <- allele_fis

allele_fiscase[,1] <- (allele_fiscase[,1]*Gamma)/((allele_fiscase[,1]*Gamma)+1-allele_fiscase[,1])

# Genotypes

# Case marker

Case_marker <- (runif(Nallele_fis*Case_number) < allele_fiscase[Case,]) +

(runif(Nallele_fis*Case_number) < allele_fiscase[Case,])

# Control 1 marker

Group1_marker <- (runif(Nallele_fis*Case_number) < allele_fis[Control_group1,]) +

(runif(Nallele_fis*Case_number) < allele_fis[Control_group1,])

# Control 2 marker

Group2_marker <- (runif(Nallele_fis*Case_number) < allele_fis[Control_group2,]) +

(runif(Nallele_fis*Case_number) < allele_fis[Control_group2,])

# Pooling sets

# Quantitative PCR

Pooling_set <- rep(1:J_set,each=900/J_set)

Case_pool <- cbind(Case,Pooling_set,Case_marker)

Case_pool <- data.frame(Case_pool)

Kappa <- runif(Nallele_fis,1,2)

Case_PCR_freq <- NULL

for (i in 1:J_set){

Each_set <- Case_marker[Pooling_set==i,]

EachSum <- colSums(Each_set)

PCR_freq <- EachSum*Kappa/(EachSum*(Kappa-1)+(900/J_set)*2)

PCR_freq <- exp(rnorm(Nallele_fis,PCR_freq,M_error))

PCR_freq <- PCR_freq / (1+PCR_freq)

Case_PCR_freq <- rbind(Case_PCR_freq,PCR_freq)

}

Group1_pool <- cbind(Control_group1,Pooling_set,Group1_marker)

Group1_pool <- data.frame(Group1_pool)

Group1_PCR_freq <- NULL

for (i in 1:J_set){

Each_set <- Group1_marker[Pooling_set==i,]

EachSum <- colSums(Each_set)

PCR_freq <- EachSum*Kappa/(EachSum*(Kappa-1)+(900/J_set)*2)

PCR_freq <- exp(rnorm(Nallele_fis,PCR_freq,M_error))

PCR_freq <- PCR_freq / (1+PCR_freq)

Group1_PCR_freq <- rbind(Group1_PCR_freq,PCR_freq)

}

Group2_pool <- cbind(Control_group2,Pooling_set,Group2_marker)

Group2_pool <- data.frame(Group2_pool)

Group2_PCR_freq <- NULL

for (i in 1:J_set){

Each_set <- Group2_marker[Pooling_set==i,]

EachSum <- colSums(Each_set)

PCR_freq <- EachSum*Kappa/(EachSum*(Kappa-1)+(900/J_set)*2)

PCR_freq <- exp(rnorm(Nallele_fis,PCR_freq,M_error))

PCR_freq <- PCR_freq / (1+PCR_freq)

Group2_PCR_freq <- rbind(Group2_PCR_freq,PCR_freq)

}

Group_PCR_freq <- (Group1_PCR_freq+Group2_PCR_freq)/2

# Statistics

D_matrix <- Case_PCR_freq-Group_PCR_freq

#D_matrix <- data.frame(D_matrix)

ChiS_total <- colSums(D_matrix)^2 / colSums(D_matrix^2)

T_statistics <- ChiS_total[1]/mean(ChiS_total[-1])

T_permutation <- NULL

for (i in 1:Permutation_times){

Per_vector <- sample(c(-1,1),rep=T,J_set,prob=c(0.5,0.5))

D_per_matrix <- D_matrix*Per_vector

ChiS_per_total <- colSums(D_per_matrix)^2 / colSums(D_per_matrix^2)

T_per_statistics <- ChiS_per_total[1]/mean(ChiS_per_total[-1])

T_permutation <- c(T_permutation,T_per_statistics)

}

P_statistics <- length(T_permutation[T_permutation>T_statistics])/length(T_permutation)

P_all <- c(P_all,P_statistics)

Count <- Count+ (P_statistics<0.05)

}
