# Supplementary material for: A Permutation Test for Oligoset DNA Pooling Studies
Source: PLoS One. 2015 Mar 12;10(3):e0119096. doi: 10.1371/journal.pone.0119096 (PMC4357378; doi:10.1371/journal.pone.0119096)

**S3 Exhibit.** Type I error rates of Huang and Lee’s [5] large-sample disequilibrium test with a total of 50 null markers (bold broken lines, $\text{σ}=0.05,\text{α}=0.05$; thin broken lines, $\text{σ}=0.05,\text{α}=0.005$; bold dotted lines, $\text{σ}=0.01,\text{α}=0.05$; thin dotted lines, $\text{σ}=0.01,\text{α}=0.005$). The horizontal bold and thin solid lines indicate the nominal α level for $\text{α}=0.05$ and $\text{α}=0.005$, respectively.


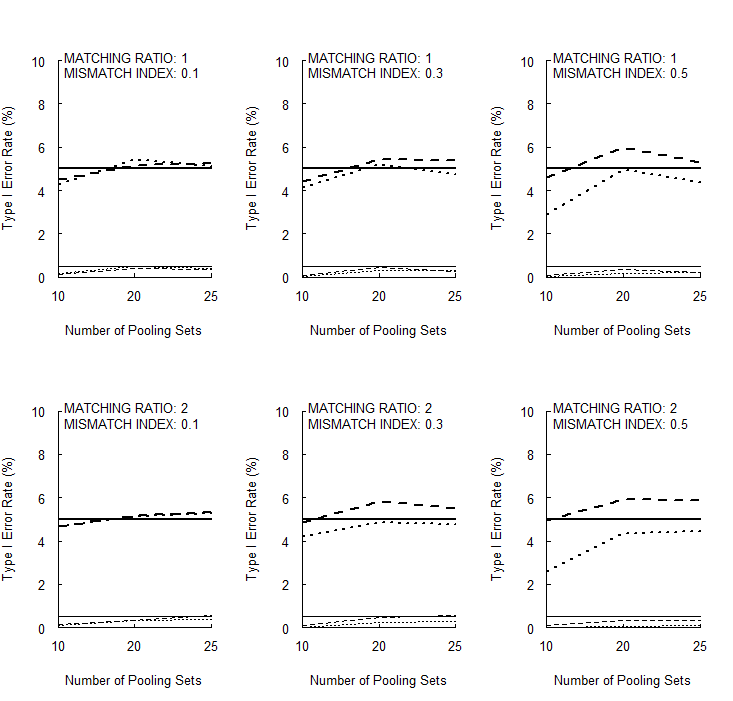

Supplement: S3 Exhibit — (DOC) [file pone.0119096.s003.doc]
